# Changing illness perceptions in patients with poorly controlled type 2 diabetes, a randomised controlled trial of a family-based intervention: protocol and pilot study

**DOI:** 10.1186/1471-2296-8-36

**Published:** 2007-06-27

**Authors:** Karen M Keogh, Patricia White, Susan M Smith, Sinead McGilloway, Tom O'Dowd, James Gibney

**Affiliations:** 1 Department of Public Health and Primary Care, Trinity College Dublin, Ireland; 2 Department of Psychology, National University of Ireland, Maynooth, Ireland; 3 Diabetes Centre, AMiNCH Hospital, Tallaght, Dublin 24, Ireland

## Abstract

**Background:**

This paper presents the pilot study and protocol for a randomised controlled trial to test the effectiveness of a psychological, family-based intervention to improve outcomes in those with poorly controlled type 2 diabetes. The intervention has been designed to change the illness perceptions of patients with poorly controlled type 2 diabetes, and their family members. It is a complex psychological intervention, developed from the Self-Regulatory Model of Illness Behaviour. The important influence the family context can have in psychological interventions and diabetes management is also recognised, by the inclusion of patients' family members.

**Methods/design:**

We aim to recruit 122 patients with persistently poorly controlled diabetes. Patients are deemed to have persistent poor control when at least two out of their last three HbA1c readings are 8.0% or over. Patients nominate a family member to participate with them, and this patient/family member dyad is randomly allocated to either the intervention or control group. Participants in the control group receive their usual care. Participants in the intervention group participate, with their family members, in three intervention sessions. Sessions one and two are delivered in the participant's home by a health psychologist. Session one takes place approximately one week after session two, with the third session, a follow-up telephone call, one week later. The intervention is based upon clarifying the illness perceptions of both the patient and the family member, examining how they influence self-management behaviours, improving the degree of similarity of patient and family member perceptions in a positive direction and developing personalized action plans to improve diabetes management.

**Discussion:**

This study is the first of its kind to incorporate the evidence from illness perceptions research into developing and applying an intervention for people with poorly controlled diabetes and their families. This study also acknowledges the important role of family members in effective diabetes care.

**Trial registration:**

ISRCTN62219234

## Background

### The importance of glycaemic control

A large body of evidence is now available showing that good glycaemic control in diabetes (as assessed by HbA1c) is associated with improved outcomes [1-6]. Current international guidelines recommend a HbA1c target level of approximately 6.0%–7.5% [7-9]. However, achieving good glycaemic control requires patients to follow a treatment regime which involves lifelong behavioural self-regulation through lifestyle changes (e.g. diet, exercise) and self-management skills (monitoring symptoms, testing blood glucose, taking medication). Many patients can have difficulties following this treatment regime[10,11] and evidence suggests only about one-third of patients with type 2 diabetes achieve glycaemic targets [12]. This has led to a call for concerted efforts to increase the proportion of patients achieving good glycaemic control [12]. It would seem prudent then, for interventions aiming to improve outcomes in diabetes, to be particularly aimed at patients having difficulties controlling their illness.

### Psychological interventions

There is growing awareness of the important role of psychosocial and behavioural factors in diabetes management [10], as highlighted by recommendations to integrate psychosocial support into routine diabetes care [8,13]. Psychological interventions to improve outcomes in diabetes have been systematically reviewed by a number of different authors, [14-16], with pooled trial results suggesting psychological interventions in diabetes reduce HbA1c by a clinically significant 1% [17]. Psychosocial interventions targeting those in poor control of their diabetes have been successful in improving glycaemic control in patients with type 1 diabetes [18-20], however there appear to be few interventions targeting those with poorly controlled type 2 diabetes.

### Illness perceptions

**Figure 1:**
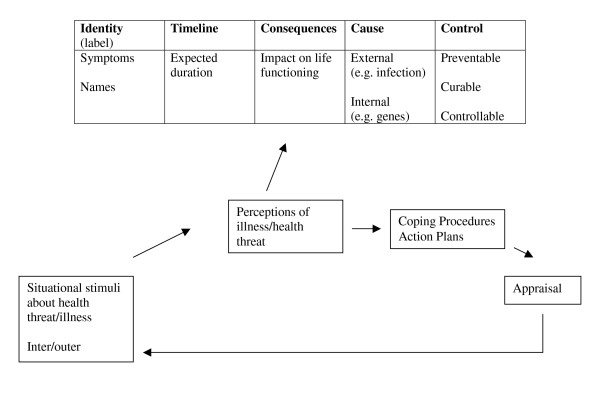
The five domains of illness perceptions [21].

One psychological approach that has been widely used in diabetes research is based on the Self-Regulatory Model of Illness Behaviour [21,22]. This approach proposes that in response to an illness, or health threat, people form their own common sense beliefs or *illness perceptions *about their illness and treatment. (The terms 'illness perceptions', 'illness representations', 'illness cognitions', and 'illness beliefs' are often used interchangeably in the literature; here the term illness perceptions is used.) These illness perceptions influence the types of health-related behaviours and coping behaviours which a patient uses for managing their illness and which may impact on disease outcomes. Research into illness perceptions [23,24] suggests they encompass five broad dimensions: identity, timeline, causes, consequences, and curability/controllability (see Figure 1: the five domains of illness perceptions). Patients' perceptions of their diabetes have been found to influence self-management behaviours [25-30] which may, in turn, impact on glycaemic control [31,32].

Patients in poor control of their diabetes have been found to have distinctly different perceptions of their illness than those in good control. A study of patients with type 2 diabetes [33] found that compared to patients in good control (HbA1c < 7), those in poor control (HbA1c > 8.5) had a stronger perception that their illness was caused by hereditary factors, reported suffering from more diabetes-related symptoms, perceived diabetes as having significantly greater impact on their lives, and reported more negative emotions in relation to their illness. Interventions focusing on changing these illness perceptions amongst patients in poor control may lead to improved illness outcomes, including better glycaemic control. A brief intervention (3 sessions)[34] designed to alter patients' perceptions about their recent MI was associated with significant positive changes in patients perceptions of their illness, as well as a significantly earlier return to work and lower rates of angina symptoms. However, there would appear to be few interventions which attempt to improve outcomes by explicitly targeting and measuring changes in illness perceptions in of type 2 diabetes.

### The role of the family

**Figure 2:**
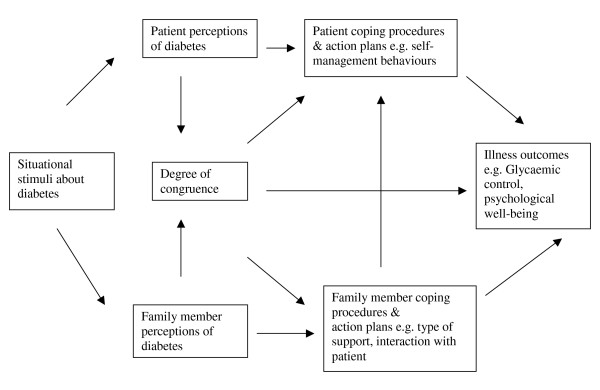
How family members may influence outcomes in diabetes.

A comprehensive understanding of how people think about, and thus manage, their illness can only be reached by taking into account the social and family context in which the thoughts were developed [35]. The possible impact of the family context on illness perceptions is particularly relevant for diabetes, as most of the self-regulatory behaviours involved in the self-management of diabetes occur at home. Evidence from a small number of studies suggests that the illness perceptions of family members may influence disease outcomes. Differences between the illness perceptions of patients with chronic illness and their spouses have been found to have a strong impact on patients' adaptive outcomes [36], while similar positive patient and spouse perceptions about the identity and consequences of MI have been found to be associated with better physical, psychological, social and sexual functioning [37]. Substantial differences have been found between family members' and patients' perceptions of type 2 diabetes [38]. Family members perceived diabetes as a more serious illness, and as having a greater impact on daily life, than those with the illness. Those with diabetes were unaware of their family member's heighten concerns and had a more relaxed approach to living with diabetes. Interventions targeting the illness perceptions of patients and families would seem a promising area for future research in view of the evidence suggesting that the degree of congruence between patient and spouse illness perceptions is related to illness outcomes. A hypothesised model by which family members illness perceptions may influence patient health outcomes in diabetes is presented in figure 2. (Figure 2: how family members may influence outcomes in diabetes.)

### Family interventions in type 2 diabetes

A number of authors have noted that the role of family factors in adult diabetes intervention research has been neglected, particularly in type 2 diabetes. [10,39-41] This is despite recent evidence suggesting that the inclusion of a family member in psychosocial interventions for chronic illness may improve illness outcomes [42-45] A recent systematic review [46 Zhang & Fisher, 2005 #516] identified only one published RCTs that included a patient's family member in an intervention for patients with type 2 diabetes [47]. This study [47] was based on a behavioural weight loss intervention, which included patients' spouses. The study found a significant weight loss in both control and family group. However, there was a significant interaction of treatment and gender, with women doing better than men when treated with their spouses as opposed to being treated alone.

This paper presents the pilot study and the protocol for a RCT that is currently underway to test the effectiveness of a family-based intervention, designed to change the illness perceptions of patients with poorly controlled type 2 diabetes and their family members.

### Trial objectives

• To examine the effects of a psychological, family-based intervention to improve biophysical, psychosocial and behavioural outcomes for patients with poorly controlled type 2 diabetes.

• To evaluate the experience of participating in the intervention.

## Methods/design

This study is a randomised controlled trial. In order to recruit a sufficiently large sample of patients with poorly controlled diabetes, participants are being recruited from diabetes specialist clinics, rather than from a primary care setting. In Ireland, the majority of patient's with type 2 diabetes in poor control of their illness are referred by their GP to a specialist clinic, and are mainly managed there. Thus, it would not have been possible to recruit the required number of participants in poor control of their diabetes from a primary care setting.

### Participants

There will be two groups of participants in the study; patients with poorly controlled type 2 diabetes and their family members. A record will be kept of all clinic attendees who do not wish to participate and their reasons for non-participation. Participants and non-participants will be compared across a number of variables (e.g. age, gender etc) to investigate any sub-group differences.

#### Inclusion and exclusion criteria

##### People with poorly controlled type 2 diabetes

People with type 2 diabetes are included in the trial if they are over 18 years of age, have fluency in English, have type 2 diabetes for more than one year, and at least two out of their last three HbA1c readings haven been 8.0% or over, in order to identify patients' with persistent poor control. (A HbA1C reading of 8% or over is a recognised value for poor metabolic control of diabetes [48].) Patients with a life-threatening physical illness (e.g. cancer, renal failure) will be excluded. Patients with a severe and enduring mental disorder (e.g. dementia, schizophrenia) as determined by the patients' clinician, will also be excluded. Patients not responsible for their own care, or those not residing in their home environment will be excluded (e.g. those in care homes, prison, in-patient hospital wards). Patients with hearing impairments will also be excluded. Patients who took part in the pilot study, and those who took part in a previous study conducted in the same diabetes clinic [33]will be excluded.

##### Family members of participants with type 2 diabetes

Participants with poorly controlled type 2 diabetes recruited to the study will be asked to nominate a family member to participate in the research with them. For the purposes of this study family members will be defined as any relative in regular contact with the person with diabetes, and who is most involved in supporting that person in the management of their illness. As most people with diabetes manage their illness themselves, the family member for this study is not a *carer*, but someone with whom the person with diabetes has a close relationship. This may include a spouse/partner, parent, grandparent, child, grandchild, siblings, or other family members. The inclusion criteria for a family member are that they must be over 18 years of age and have no medical history of type 1 or type 2 diabetes.

### Screening eligibility

**Figure 3:**
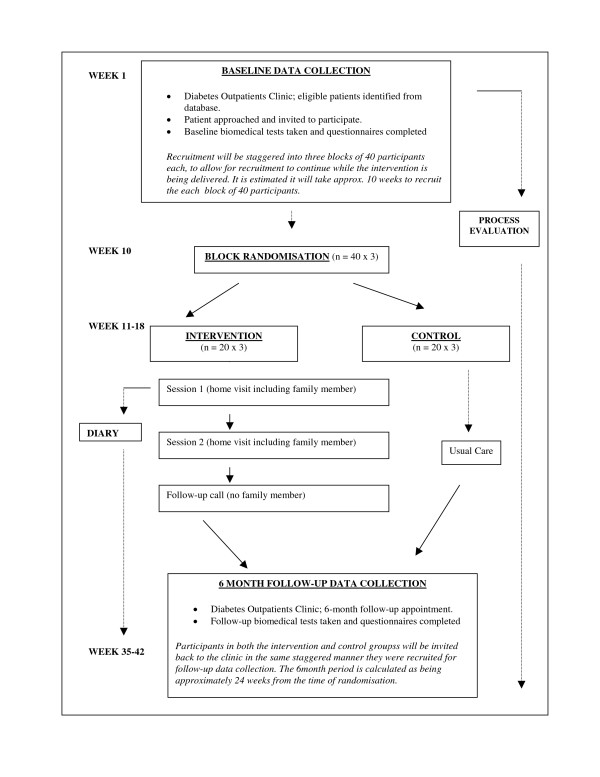
Flow Chart of RCT.

Potential participants will be recruited from two diabetes specialist clinics at the AMNCH Hospital in Tallaght in Dublin. All type 2 patients over 18 years, with at least two of their last three HbA1c readings of 8.0% or greater (eligible participants), will be identified through the auditing facility of the "Diamond" computer database (n~ 3560 type 2). Prior to each clinic a list of eligible patients attending the clinic will be generated by the Diamond database, who will subsequently be asked to participate in the study (see Figure 3: Flowchart of RCT).

### Baseline assessment

During the twice-weekly out-patients clinics eligible patients will be approached by the researcher, given information about the study and invited to participate. On agreement, they will be asked provide their written informed consent, and to complete the baseline questionnaires with the researcher (this will take on average fifteen minutes). These questionnaires consist of demographic questions, the brief-illness perceptions questionnaire [49], the well-being 12 scale [50], the diabetes management self-efficacy scale [51], the diabetes family behaviour checklist [52]and the summary of diabetes self-care daily activities [53]). At this appointment time their HbA1c, blood pressure, and body-mass index will also be taken. The person with diabetes will also be given a questionnaire pack containing the family member measures, and asked to ensure their nominated family member completes the questionnaires and returns them by post to the researcher, prior to commencement of the intervention. The family member questionnaire include demographic questions, and adapted versions of the brief illness perceptions questionnaire [49]and the diabetes family behaviour checklist [52].

### Outcome assessment

At six months post-intervention (approximately 24 weeks from the time of randomisation), control and intervention participants will be invited back to the diabetes clinic in the same staggered manner as they were recruited. At this time point, HbA1c, blood pressure, cholesterol and body-mass index will be taken again and recorded. The baseline questionnaires will also be re-administered. In addition, a record will be kept of participation rates throughout the intervention and reasons for non-participation where possible. Participants will again be given a questionnaire pack containing the family member measures, and asked to ensure that their nominated family member completes the questionnaires and returns them to the researcher by post.

### Randomisation

After consent and baseline data have been collected from the first 40 patients, they will be randomized into intervention and control groups by using computer generated random number tables. The main investigators will not be involved in the randomisation procedure, which will be carried out by an independent expert. The use of block randomisation means that the intervention can be delivered in a staggered manner, with some participants beginning the intervention while further recruitment continues. When the next 40 patients are recruited, they will then be randomly allocated to intervention or control groups, and so on, until 122 patients are included in the trial. Due to the psychological nature of the intervention, it is not possible for the investigator delivering the intervention to be blind to participants' treatment allocation group.

### Intervention

The intervention is a complex psychological intervention designed to change the illness perceptions of patients with type 2 diabetes and their family members. The starting point for the intervention is the illness perceptions models of diabetes held by the patient and the family member and the degree of similarity between these models. The intervention is based upon clarifying the five illness perception dimensions of both the patient and the family member in relation to diabetes, examining how they influence self-management behaviours, and developing personalized action plans. The intervention will be tailored to each individual patient and family member; thus, the exact content of each session will depend upon the individual illness perceptions of the patient and family member, and the degree of congruency between these perceptions. In essence, the intervention is designed to change any inaccurate and/or negative illness perceptions which the patient or family member may have, and improve the degree of congruence of patient and family member perceptions, in a positive direction. A sample case study is presented in Table 1 to illustrate how the intervention might work (Table 1: sample case study for intervention). A more detailed description of the intervention can be found in the intervention manual (see additional file 1)

**Table 1:** Sample case study for intervention

FC, male mid 50's, with poorly controlled type 2 diabetes. He says he understands very little about his diabetes (illness coherence), but that he feels it has a huge impact on his life e.g. he hates taking the medication, he is tired all the time etc (consequences). FC doesn't believe lifestyle factors are important in controlling his illness (control), because he believes the causes of the illness are purely genetic (he believes he inherited the illness from his mother – causal). His wife, MC, believes that while she does not understand the diabetes, her husband understands his diabetes very well (coherence), but that it has very little impact or effect on his life (consequences); She also thinks that he is over-reacting when he complains about it. She believes his diabetes was caused by stress (cause), and if he stopped working so much and took more time to relax, his condition would improve (control). She also does not recognise the importance of lifestyle factors such as diet and exercise for controlling diabetes (control), because she thinks the illness is stress-related and continues to prepare high-fat, high-sugar meals for her husband.
The intervention sessions with this couple could be tailored to focus on clarifying the causal dimension of illness perceptions of both participants, by focusing on the risk factors associated with developing type 2 diabetes. In particular, the importance of lifestyle factors in controlling the illness could be emphasised, and attempts to improve the patient's level of personal control over the illness. The intervention could also focus on highlighting and resolving differences between the patient's and family member's illness perceptions, such as the discrepancy between the perceived consequences and levels of understanding between the patient and his wife. A written, personalised action plan to improve control of the patient's diabetes could then be developed in collaboration with the patient and his wife. This could include, for example, an agreement for the patient and his wife to take time to go out walking together three times a week, to reduce the levels of fatty and sugary foods consumed etc.

The intervention that is the focus of this study is relatively brief, in view of recent research suggesting that briefer psychosocial interventions can be more easily integrated into routine care. Due to the brief nature of the intervention and its emphasis on behaviour change, techniques from brief motivational interviewing [54] will be used to deliver the intervention. These techniques have been successfully used in other brief interventions of this type [55,56]. The intervention will consist of three sessions delivered on a weekly basis, the first two of which will take place in the patient's home with their family member, and the last one of which consists of a phone call to the person with diabetes. Sessions will be delivered by a health psychologist who is trained in motivational interviewing techniques. Each session will last approximately 40 minutes.

### Sample size and rate of recruitment

Taking HbA1c and Diabetes Well-being as primary outcomes, a total sample size of 76 and 86 respectively, were calculated. This was using 80% power to detect a significant *absolute *change of 0.9% in glycaemic control (this absolute change has been related to clinical outcomes in the UK prospective diabetes study, [3] and of 3 points in the Diabetes Well-being Scale-12. These calculations also allow for an anticipated "Hawthorn effect" relating to an improvement of 20% for those in the control group, by virtue of the fact that they are participating in the research. Taking the larger number of86 participants (43 inintervention and 43 in control group), a final total number of 122 participants (61 in each group) is needed to ensure at least a 70% final response rate is met. Previous research in the diabetes clinic [33] has shown that it is possible for one researcher to see approximately two to three eligible patients per clinic. Thus, it is estimated that it will take approximately 8–10 weeks to recruit the required 40 participants for the first block for randomisation.

### Qualitative component

Participants with type 2 diabetes (in the intervention group only) will be asked to complete a brief diary on a weekly basis for the first six weeks and every second week for the remaining eighteen weeks. Diaries will be structured to allow for the examination of the intervention and the process of change over time. In order to maximize compliance to filling in the diary, weekly/fornightly text messages will be sent to remind participants to fill in their diary. A computer program will be used to send text messages to a large number of mobile phones at regular intervals.

### Quality assurance

The process evaluation component of this RCT will run alongside data collection and intervention delivery. The use of both qualitative and quantitative data provides the strongest evidence for process evaluation [57,58]. Therefore, qualitative and quantitative process evaluation data will be collected in this study, focusing on aspects of intervention implementation, participant experience of receipt of the intervention, and the influence of contextual/setting factors. See Table 2 for an outline of the process evaluation questions and data collection tools (Table 2: process evaluation components).

**Table 2:** Process evaluation components

Process Evaluation Questions	Data Collection Tool	Source	Trial Stage
***Implementation*** **Was the intervention properly delivered?** **Treatment fidelity to different components.** **Monitor dose/participant exposure to intervention components**	1.10% of sessions randomly selected to be taped and analysed by independent expert in illness perceptions and motivational interviewing. Tapes analysed qualitatively and quantitatively using MI & IP checklists.	Randomly selected sub-sample of intervention participant's.	Collecting during intervention delivery. Analysed post-intervention

***Receipt*** **1.Participants views of the intervention and partaking in the RCT.**	1.Open-ended questionnaires for all participants in control and intervention groups	1. All participants	1. Collected at follow-up data collection
**2.Appropriateness of use of intervention and techniques for type 2 diabetes**	2. Focus groups with sub-sample of intervention participants	2. Sub-sample of intervention participant's.	2. Collection post-follow-up

***Contextual factors*** **What was the effect of various setting/contextual factors. e.g. interruptions during session, family dynamic, mood, etc.**	Structured field notes questionnaire (e.g. how long each session, where in home delivered, interruptions, dynamic, etc)	Interventionist	Collecting during intervention delivery. Analysed post-intervention
**Sub-groups variations (e.g. family member involved)**	Interventionist observations – field diary		

### Analysis

#### Quantitative analysis

SPSS Version 13.01 for Windows will be used in the analysis. Differences in biomedical, psychosocial and illness-specific outcomes between the intervention and control group will be analyzed for the two time-points (baseline and 6 months post- intervention). Further statistical analysis will be based on repeated measures analysis of variance. Statistical significance will be taken at the 5% level for primary outcomes, and at 1% level for secondary outcomes.

#### Qualitative analysis

Qualitative data will be analysed using phenomenological techniques. Both content analysis and thematic analysis will be applied to the data

### Trial organisation and management

The trial is being managed by the Department of Public Health and Primary Care, Trinity College Dublin, and the Diabetes Centre, Adelaide and Meath incorporating the National Children's Hospital, Tallaght, Dublin. Ethical approval was obtained from the Joint Research Ethics Committee of St. James's Hospital and the Federated Dublin Voluntary Hospitals

## Pilot study

**Figure 4:**
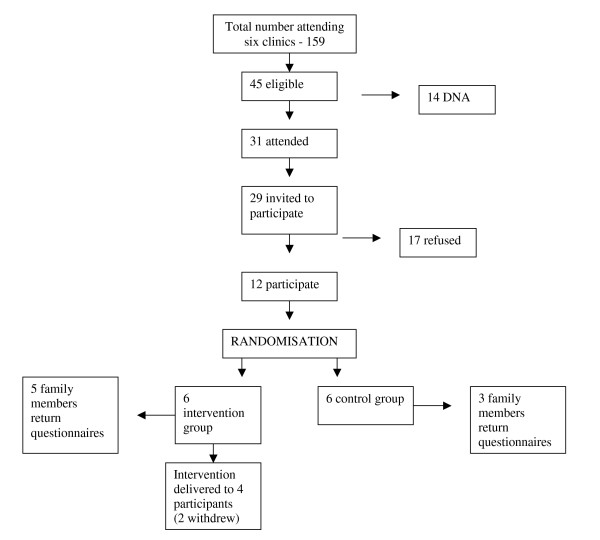
Flowchart of recruitment of pilot participants.

Participants for the pilot study were recruited from six weekly clinics in the Diabetes Centre, during the summer of 2006. Twelve eligible patients agreed to participate in the pilot and completed baseline assessment. Participants were divided into intervention and control groupss; six in the intervention and six in the control. Two participants in the intervention group, and one from the control group, withdrew from the study, leaving nine participants in the pilot study, four of which completed the intervention sessions. Pilot participants were not recalled for outcome assessment, since the small sample size would not allow for statistical analysis. A flow-chart of the recruitment process for the pilot study is shown in figure. 4 (Figure 4: Flowchart of recruitment of pilot participants).

The pilot study showed that the procedures, measures, and delivery of the intervention worked well and recruitment began for the main study in October 2006. However, following the pilot study, it was considered necessary to change one of the measures to be used in the RCT. Following feedback from some of the participants showing that the original self-efficacy measure was quite difficult for them to understand; this measure was replaced by the Diabetes Management Self-Efficacy Scale[51]. The rate of recruitment for the pilot was also slower than anticipated. To ensure sufficient numbers of participants were recruited for the main trial, it was decided it would be necessary to recruit participants for the main trial from an additional weekly clinic.

## Discussion

This trial aims to assess the effectiveness of a family-based, psychological intervention to improve outcomes in those with poorly-controlled type 2 diabetes. The intervention recognises the important role of family members in effective diabetes care, and it is the first of its kind to adapt evidence from the illness perceptions research to an intervention for people with poorly-controlled diabetes and their families.

The intervention is based on a clearly specified theoretical framework; Leventhal's Self-Regulatory Model of Illness Behaviour [21,22]. There is much empirical evidence showing that the concepts of this model are related to illness outcomes in diabetes, and illness perception interventions in various disease populations have also been successful in changing illness perceptions and improving illness outcomes [34].

The study is further located within three main theoretical frameworks outlined by Matire and colleagues [44], including the bio-psychosocial model e.g. [59], martial and family system framework e.g[60], and family care-giving and receiving model e.g. [61], with some evidence to suggest family members own illness perceptions of the patients illness can influence outcomes. It has been proposed that family members may influence outcomes in physical health by means of a psychophysiological and/or health behaviour pathway, [42,43]. This intervention targets both of these pathways insofar as it attempts to change negative illness perceptions and increase the degree of similarity of patient and family member perceptions. These may in turn directly impact upon self-management behaviours (e.g. through increased self-efficacy, support for diabetes-specific activities), and indirectly improve the nature and quality of family functioning and interactions (e.g. through increased understanding, more general support).

This trial includes a combination of both process indicators and outcome measures as recommended by a number of authors [57,58,62], The process evaluation component will provide detailed information on how the intervention was delivered and received, which will allow for increased generalisability of results, whilst also ensuring quality through the assessment of treatment fidelity. A primary aim of the process evaluation is to establish *why *the intervention achieved its results. Thus, if the intervention has little or no impact on outcomes, the process evaluation data should uncover whether this was due to inadequate design (a failure of concept/theory), poor delivery (implementation failure) and/or or other factors.

## Competing interests

The author(s) declare that they have no competing interests.

## Authors' contributions

PW & SS conceived the development of the intervention. PW, SS and KK developed the intervention. All authors developed the trial protocol and contributed to drafting the manuscript. SS is the principal investigator. KK manages the running of the trial.

## Pre-publication history

The pre-publication history for this paper can be accessed here:



## Supplementary Material

Additional File 1 Intervention Manual Click here for file
