# Supplementary material for: Changing illness perceptions in patients with poorly controlled type 2 diabetes, a randomised controlled trial of a family-based intervention: protocol and pilot study
Source: BMC Fam Pract. 2007 Jun 27;8:36. doi: 10.1186/1471-2296-8-36 (PMC1919379; doi:10.1186/1471-2296-8-36)
Supplement: Additional File 1 — Intervention Manual [file 1471-2296-8-36-S1.doc]

**INTERVENTION MANUAL**

**OUTLINE OF SESSIONS**

## **Session 1** Includes person with type 2 diabetes and his/her family member.

## Delivered in the participants' home.

## **Opening**

*Use MI strategies re: opening*

- **Illness coherence**
  - Identify levels of understanding from BIPQ
  - Explanations of pathophysiology and terminology
- **Identity**
  - Identify how often experience symptoms from BIPQ
  - Explain/discuss symptoms of poorly controlled diabetes
- **Casual beliefs**
  - Identify causal beliefs from IPQ
  - Challenge misconceptions and broaden causal beliefs
- **Emotional representations**
  - Identify how much diabetes affects emotions

*Use MI Strategies re: exchanging information*

## **Session 2** – Includes person with type 2 diabetes and his/her family member.

## Delivered in the participants' home.

- **Consequences**
  - Identify causes from BIPQ
  - Challenge misconceptions and link with current behaviour
- **Timeline**
  - Identify acute/chronic and cyclical/stable illness perceptions
  - Link with consequences and challenge myths/misconceptions
- **Control**
  - Identify personal and treatment control beliefs from BIPQ
  - Use control beliefs to develop personal plan by identifying areas of diabetes management that *can* be controlled and that need improvement

*Use MI Strategies re: exchanging information*

⇓

- Develop **Written Personalised Action Plan**; improving control and minimise complications through agreed goals

*Use MI strategies: Negotiating a change plan*

## **Session 3** –phone call with person with type 2 diabetes only.

1. Review action plan and discuss potential barriers to proposed changes. Amend plan as necessary
2. Discuss any further concerns
3. Use motivational interviewing strategies discussed previously, through-out the session where appropriate.

**MOTIVATIONAL INTERVIEWING GENERAL PRINCIPLES**

Through out the sessions it may be helpful to keep in mind the four general principles of motivational interviewing;

1. Expressing empathy
2. Rolling with resistance
3. Developing discrepancy
4. Building self-efficacy.

These principles, and their corresponding techniques, have not be given a fixed point for being conducted in sessions, rather they may be useful with different participants at different times, as the interventionist feels it is appropriate.

**SESSION ONE**

**OPENING**

*Use MI strategy re: Opening Structure*

It can be useful from the outset to provide participants with a simple and brief structuring statement. This can help participants feel relaxed and comfortable with the session, and allay any fears or concerns. Some elements to incorporate into an opening statement include;

- The amount of time and number of sessions
- An explanation of the interventionists goals and role
- A description of the clients role in the sessions
- Any details that must be attended to
- Finish with an open ended question

EXAMPLE OPENING STATEMENT TO PERSON WITH DIABETES;

*“We have about 45 minutes together now, and another session of 45 minutes next week and I’ll also be contacting you twice by telephone. I’ll spend most of the time listening so I can get a better understanding of how you see your diabetes management, and your issues and concerns. You’ll probably have some hopes about what will and won’t happen here, and I’d like to here about those. I’ll probably ask you some more specific information towards the end of the session, but for now why don’t you start by telling me what’s on your mind?”*

OPENING STATEMENT TO FAMILY MEMBER

It is important from the start to be clear about the role of the family member. The focus of the session is on the person with diabetes. The significant other will be included as a *participant* in session, the goal is not to target couple interactions or conduct motivational interviewing with the family member. Thus, the family members role will be in sharing information collaborate in the planning and development of goals and how to achieve them, and in general, to help promote the client’s commitment to change. Participation of the family member may also be useful in revealing “hidden” information, and enhance the truthfulness of the clients talk. An example of an opening statement to a family member might be;

*“It’s great that you’re able to be here with us. I hope that you will feel comfortable in playing an active role in the session, and discuss your own concerns about your family member’s diabetes, and your own hopes about what will happen here.”*

***MOTIVATIONAL INTERVIEWING STRATEGY: EXCHANGING INFORMATION***

For the remainder of session one, and part of session two, the predominant MI strategy used will be related to the exchange of information. There are a number of steps relating to the exchange of information using motivation interviewing techniques:

1. Elicit from the participant what s/he already knows; e.g.

*“Could you tell me what you know about your illness?”*

*“What is your own understanding of Type 2 diabetes?”*

1. Find out what else the participant would like to know? e.g.

*“Is there anything else about your illness you would like to know more about?”*

*“Is there any aspects of your illness you feel a bit unsure about?”*

1. Ask permission to give information e.g.

*“I have some information you might find useful, if you would like to hear it?”*

*“I could give you some more information on that if you would like me to?”*

1. Provide the facts neutrally e.g.

*“There are some scientific studies that show…what do you make of that?”*

*“It’s a medical fact that…but what is important to me what you think about this?”*

1. Elicit from the participant how they might apply the facts to their own situation

*“How might that apply to you?”*

***“****Would any of that be relevant to you and your situation?”*

1. Include the family member – may help uncover “hidden” information and develop discrepancy

*“What is your understanding of your family member’s diabetes?*

*“Are there aspects of your family member’s diabetes that you are unsure about or would like to know more about?”*

*“What are some of your concerns about your family members’ diabetes?”*

***“****How do you see the situation?”*

This strategy can be adapted and applied to following discussions of illness perceptions on the concepts of illness coherence, identity, causal beliefs and emotional representations in session one, and consequences, timeline and control beliefs in session two.

**ILLNESS COHERENCE**

*Use MI strategy re: exchanging information*

Participants’ levels of understanding of their type 2 diabetes will be identified from the question on the B-IPQ *“How well do you feel you understand your type 2 diabetes?”* Answers to the question are scored on a scale from 0 (don’t understand at all) to 10 (understand very clearly). An explanation of the pathophysiology and terminology of type 2 diabetes will be individually tailored to each participant, based on their score, e.g. a score of 0 would require a more in-depth discussion than a score of 10. The exact content of the discussion will vary by individual, but may include a discussion on:

- **What is diabetes?** (using diagrams to provide a concrete image of the illness)
- **Continuum of diabetes and Treatment** (Brief explanation of differing types of treatment for type 2 diabetes and its progressive nature)
- **Controlling/self-managing diabetes**
  - **Lifestyle Factors**
    - **Diet**
    - **Exercise**
  - **Medication**
    - **OHAs**
    - **Insulin in Type 2 diabetes**
  - **Blood glucose monitoring**

**IDENTITY**

*Use MI strategy re: exchanging information*

How often participants experience symptoms of their type 2 diabetes will be identified from the question on the B-IPQ *“How much do you experience symptoms from your type 2 diabetes?”* Answers to the question are scored on a scale from 0 (no symptoms at all) to 10 (many severe symptoms). A discussion of the symptoms of poorly controlled diabetes will be based on each individuals score, and how these symptoms can be reduced by improved metabolic control. Symptoms of poorly controlled diabetes that may be discussed include;

- **Hypoglycaemia and hyperglycaemia**

**CAUSAL BELIEFS**

*Use MI strategy re: exchanging information*

The causes people attribute to their diabetes will be identified from the last question on the B-IPQ, which asks people to *“Please list in rank order the three most important factors that you believe caused your illness.*  *The most important causes for me:-“* The discussion will be focused on the answers participants give to this question, and depending on these answers may challenge any misconceptions (e.g. participant believes only cause is hereditary so there is no point in doing anything, or diabetes is caused by eating too much sugar). Where relevant, the discussion will try and broaden the participants’ causal model to include the importance of lifestyle factors (e.g. diet and exercise), which could be used as a source of behaviour change.

**EMOTIONAL REPRESENTATIONS**

The question on the B-IPQ *“How much does your illness affect you emotionally? E.g. does it make you angry, scared, upset or depressed?”* will be used to assess how much type 2 diabetes affects participants’ emotions. This question is scored on a scale of 0 (not at all affected emotionally) to 10 (extremely affected emotionally). The discussion regarding emotions will not focus on changing these emotions per se, but rather the main focus will be on how participants’ feelings about their diabetes impact on their self-care behaviour and diabetes management. (e.g. someone is so angry about their illness they won’t take their medication or someone feels that their diabetes is taking over their life and they can do nothing to control it).

**THE ROLE OF THE FAMILY MEMBER IN ILLNESS PERCEPTIONS**

During the discussion on the person with diabetes illness perceptions (session 1and 2), it may be useful to also refer back to the family members’ scores on the B-IPQ, and bring in their perceptions of their family members diabetes. This includes the family member in the discussion, and may also be useful in highlighting areas of discrepancy between their perceptions of diabetes, and the person with diabetes own illness perceptions. For example, the family member may believe the person with diabetes is very knowledgeable about their illness, whereas the person with diabetes may feel they know very little. After area’s of dissimilarity have been identified, the session may focus on improving the degree of congruence, in a positive direction, between patient and family members perceptions of diabetes.

**SESSION TWO**

**CONSEQUENCES**

*Use MI Strategies re: exchanging information*

Participants perceptions of the consequences of their type 2 diabetes will be identified from the question on the B-IPQ *“How much does your illness affect your life?”* This question is scored on a scale of 0 (no affect at all) to 10 (severely affects my life). A discussion on the consequences of type 2 diabetes on the participants’ life will be based on individual participants’ answers to the above question. In general, the discussion will focus on challenging any misconceptions participants have about the consequences of their illness, and linking these beliefs with current behaviour, e.g. how participants’ beliefs about diabetes consequences impact on their daily adherence to self-care behaviours.

**TIMELINE**

*Use MI strategies re: exchanging information*

The B-IPQ assesses participants’ timeline beliefs about their type 2 diabetes by asking the question *“How long do you think your illness will continue?”* This question is scored on a scale of 0 (a very short time) to 10 (forever). Acute/chronic and cyclical/stable illness perceptions will be identified through discussion with the participants. Again, any misconceptions will be challenge (e.g. diabetes comes and goes in cycles). The discussion will link the timeline aspects back to the discussion on consequences and the influence of current behaviour. (e.g. type 2 diabetes may “come and go” because at times diabetes symptoms may be better due to improved metabolic control).

**CONTROL**

*Use MI strategies re: exchanging information*

Personal control beliefs about type 2 diabetes will be identified from the B-IPQ from the question *“How much control do you feel you have over your illness”* This question is scored on a scale from 0 (absolutely no control) to 10 (extreme amount of control). Beliefs about treatment control will be identified from the question on the B-IPQ *“How much do you think your treatment can help your illness?”* This question is also scored on a scale of 0 (not at all) to 10 (extremely helpful). Participants’ beliefs about personal and treatment control will be discussed, and used to identify areas of diabetes self-management that participants can control, and areas that may need improvement (e.g. feel very confident about taking medication, but less so about exercising).

**DEVELOPING PERSONALISED WRITTED ACTION PLAN**

*Use MI Strategies re: negotiating a change plan*

Areas of diabetes self-management that need to be improved should arise from the discussion on control beliefs. Stemming from this will be the development of a written personalised action plan. This plan will be negotiated with participants’ using a process of shared decision making and MI strategies. It involves a series of steps.

***MOTIVATIONAL INTERVIEWING STRATEGY: NEGOTIATING A CHANGE PLAN***

1. **Setting goals**

The first step in instigating change is to have clear goals to work towards. Ask the participants what these goals are (direct questions to both person with type 2 diabetes and their family member);

*“What is it that you want to change?”*

*“Lets take things one step at time. What do you think is the first step?”*

The participants’ goals may not correspond to the interventionist’s, it is far better to start with the goals that the client is eager to make progress towards. Let the participant chose what area they feel they can, and want to, start to work on. Letting the participant chose which area they feel they can work on and succeed at improves self-efficacy and a sense of personal control. There may also be multiple goal also with multiple and interrelated areas of concern. It may be necessary to prioritise which are most urgent or important.

1. **Considering change options**

The next step is to consider possible methods for achieving chosen goals.

*Use MI strategies re: brain storming and “menu” options*

BRAIN STORMING

Involve the person with diabetes and the family member directly in the process of brainstorming and evaluating possible change strategies. There are almost always multiple ways to achieve a behaviour change goal. It can be useful to use a creative brain-storming session, in which evaluation is temporarily blocked, to generate a range of options, even far-fetched ones. This can be a pooling of the person with type 2 diabetes ideas, the family members ideas, and the interventionists. This can help develop personal control and self-efficacy, as the participant can suggest ideas without being blocked by any shortcomings and the interventionist can suggest ideas with out meeting resistance.

“MENU” OPTIONS

It is also often useful to provide a “menu” of strategies, rather than one at a time. The participants’ task then becomes one of choosing, rather than refuting.

1. **Arriving at a plan**

This discussion leads directly to negotiation of a change plan. Elicit this plan by having the participant voice it in person, by asking;

*“So what is it specifically that you plan to do?”*

*“How will you go about it?”*

At this stage introduce the *Change Plan Worksheet* (see Appendix 1 p.14) that the person with diabetes can fill out, this is their written personalised action plan. On the basis of this discussion, summarise the plan that has been developed, using “you” language. For example;

*“Let me see if I can accurately summarise where you are then. What you want to do is work on controlling your diabetes better, through diet and exercise. You plan to go walking for at least 30minutes most days. And you and your family member have agreed to make healthy meals together, based on the guidelines for healthy eating that I’ve given you.”*

1. **Eliciting commitment**

The plan summary brings you to the point of commitment. The simplest way to get this is to ask;

*“Is this what you want to do?”*

Getting a “yes” may require a few more steps, by exploring reluctance and ambivalence using previously discussed strategies for resolving ambivalence.

Making the change plan public can enhance commitment, this can be done by making the family member a “witness”, and acknowledging that the commitment to change is made with the family member’s consent and knowledge and support.

**THE ROLE OF THE FAMILY MEMBER IN NEGOTIATING A CHANGE PLAN**

The family member can be very actively included in the negotiating of the change plan. They can participate in the *setting of goals*, by indicating what goals they would like to see the person with diabetes work towards. The can contribute to the *considering change options* discussion by taking part in the brain-storming session and coming up with ideas that can help the person with diabetes achieve their goals, and contributing these to the *change plan*. They also act as a “witness” to the person with diabetes *commitment* to change.

Most importantly, throughout this process the family member can contribute ways in which they themselves can support the person with diabetes in managing their illness and achieving better metabolic control.

**SESSION 3**

**Outline –** with person with type 2 diabetes only, over the phone

1. Review action plan and discuss potential barriers to proposed changes. Amend plan as necessary
2. Discuss any further concerns
3. Use motivational interviewing strategies discussed previously, through-out the session where appropriate.

**MOTIVATIONAL INTERVIEWING TECHNIQUES**

Millner and Rollinick (2002) write that *“…we have found ourselves placing less emphasis on techniques of motivational interviewing and ever greater emphasis on the fundamental spirit that underlies it”* (Millner & Rollnick, 2002, p. 33). Through the sessions it may be useful and appropriate to follow the four general principles of motivational interviewing; expressing empathy, developing discrepancy, rolling with resistance and supporting and building self-efficacy. These principles are not formally included at a set point during sessions, because as Millner and Rollick point out, they are more a “spirit” than formal techniques. They may be useful at various different time-points, with various participants, as the interventionist feels it is appropriate.

**EXPRESSING EMPATHY**

**ROLLING WITH RESISTANCE**

Resistance responses to change are normal. The key point is how the interventionist responds to resistance. In motivational interviewing the goal is to diminish resistance by “rolling” with it, and avoiding arguing for change. General methods for “rolling with resistance" include;

1. **Reflections –** respond to resistance with non-resistance

- Simple reflections
- Amplified reflections
- Double-sided reflections

1. **Shifting focus** (shift attention away from problem area)
2. **Reframing** (recast participants information in a new light)
3. **Agreeing with a twist** (offer initial agreement with change of direction)
4. **Emphasising personal choice and control** (avoids “psychological resistance”)

**DEVELOPING DISCREPANCY AND ELICITNG CHANGE TALK**

When the participants’ perceived importance of change is low, the client’s motivation to change and their perception of the importance of change may be enhanced by developing discrepancy. Discrepancy can be considered as an awareness of a difference between where one is and where one wants to be. It is discrepancy that underlies the perceived importance of change; without discrepancy there is no motivation, and the larger the perceived difference, the greater the importance of change.

The first step is to develop ambivalence by developing discrepancy between the actual present and the desired future. Change is facilitated by communicating in a way that elicits the person’s own reasons for and advantages of change. The interventionist can evoke change talk using some of the following methods;

1. **Asking open-ended evocative questions e.g.**

- *“What worries you about your current situation?”*
- *“I can see you’re feeling stuck at the moment. What’s going to have to change?”*

1. **Using the importance ruler e.g.**

- *“How important would you say it is for you to manage your diabetes well? On a scale from 0 to 10, where 0 is not important at all and 10 is extremely important, where would you say you are?*

1. **Exploring the pro’s and con’s of change e.g.**

- *“Why don’t you tell me what you like about the way things are at the moment?”*
- *“What are the advantages of your current situation?”*

1. **Querying extremes e.g.**

- *“Suppose you continue on as you have been, without changing, What do you imagine are the worst things that could happen?”*
- *“What might be the best results you could imagine if you make a change?”*

1. **Looking back or looking forward e.g.**

- *Do you remember a time when you managed your diabetes well? What has changed?”*
- *“How would you like things to turn out for you 10 years from now?”*

**BUILDING PERSONAL CONTROL AND SELF-EFFICACY**

Generally, people cannot be ready to change until they perceive both that they want to (importance, discussed above) and that they are able to (confidence/self-efficacy). When a participant wants to change (importance is high) but there confidence in doing so successfully is low, there are a number of motivation interviewing techniques that can be used to improve low-self efficacy;

1. **Scale questions** (about confidence for change) **e.g.**
   - *“How confident would you say you are that if you decided to improve the control of your diabetes, you could do it? On the same scale fro 0 to 10, where 0 is not at all confident, and 10 is extremely confident, where would you say you are?*
   - *“Why are you at____ not ____?”*
   - *“What would it take for you to go from ___ to____ (a higher number)?”*
2. **Reframing** (Discuss previous less successful attempts to change in a positive light).
   - *“What have you learned from previous attempts to control your diabetes, about what works, or doesn’t work for you?”*
   - *“It sounds to me like you’re an expert in what doesn’t work for you”*
3. **Past successes**
   - *“When in your life have you made up your mind to do something, and did it?”*
   - *“What obstacles were there and how did you overcome them?”*

4.  **Personal strengths and support**

- *“What is there about you, what strong points do you have that would help you to succeed in controlling your diabetes better?”*
- *“Is there anything you know of that has worked for other people?”*

**Appendix 1 – Change Plan Work Sheet**

The changes I want to make are:

The most important reasons why I want to make these changes are:

The steps I plan to take in changing are:

The ways my family member __________________ can help me are:

I will know what my plan is working if:

Some things that could interfere with my plan are:
